# Neurological complications in COVID-19 – a diagnostic challenge

**DOI:** 10.25122/jml-2021-0045

**Published:** 2021

**Authors:** Vitalie Vacaras, Sorina Frunze, Adrian Mihai Cordos

**Affiliations:** 1. Neurology Department, Iuliu Hatieganu University of Medicine and Pharmacy, Cluj-Napoca, Romania; 2. Neurology Department, Cluj-Napoca County Emergency Hospital, Cluj-Napoca, Romania

**Keywords:** neurology, COVID-19, stroke, subarachnoid hemorrhage, cardiovascular medicine, ACE2 – angiotensin converting enzyme II, BBB – blood-brain barrier, BMI – body mass index, BP – blood pressure, CNS – central nervous system, COVID-19 – coronavirus disease, CRP – C-reactive protein, CSF – cerebrospinal fluid, CT – Computer tomography, CTA – Computer tomography angiography, CVT – cerebral venous thrombosis, ECG – electrocardiogram, Holter ECG – Holter electrocardiogram, HR – heart rate, I.V. – intravenous, MRA – Magnetic resonance angiography, LMWH – low molecular weight heparin, MRC scale – Medical Research Council's scale, MRI – Magnetic resonance imaging, PACNS – primary angiitis of the central nervous system, PCA – posterior cerebral artery, PCoA – posterior communicating artery, PCR – polymerase chain reaction, RCVS – reversible cerebral vasoconstriction syndrome, RT-PCR – real-time polymerase chain reaction, RR – reference range, SAH – subarachnoid hemorrhage, SARS-CoV-2 – severe acute respiratory syndrome coronavirus 2, UL – upper limit

## Abstract

With the exponential growth of COVID-19 cases, the neurological complications reported during or after the infection became more common. There is limited knowledge regarding the pathophysiological mechanisms that are responsible for these complications. Recent data provides compelling evidence for the neurotropic nature of SARS-CoV-2, based on neurological manifestations reported during the current pandemic, as well as on previous experience with other coronaviruses. We present the case of a patient who developed headaches, motor deficit and dysphasia after respiratory COVID-19. Imaging tests showed heterogeneous central nervous system lesions (multiple subarachnoid hemorrhages and two ischemic strokes). Given the plethora of atypical neurological complications of COVID-19 described in the current literature, establishing a positive diagnosis and deciding on a treatment plan proved to be particularly challenging. We set to discuss some of the possible pathologies, hypothesized to be associated with COVID-19, that could lead to concomitant neurological lesions, similar to those noticed in our patient.

## Introduction

In December 2019, several health facilities in China reported clusters of patients presenting with pneumonia of unknown etiology, which soon led to the discovery of a novel coronavirus named 2019-nCoV [1, 2]. The virus was later given the official name, Severe Acute Respiratory Syndrome Coronavirus-2 (SARS-CoV-2), and the disease it was responsible for was named Coronavirus Disease 2019 (COVID-19) [3]. On March 11, 2020, after the virus spread to 114 countries and the total number of cases reached 118.000, COVID-19 was declared a pandemic [4].

A retrospective, single-center case series of 138 hospitalized patients with confirmed COVID-19 pneumonia, set in Wuhan, China, reported in 2020 that the most common symptoms at illness onset were fever, fatigue, dry cough, myalgia, and dyspnea. Less common symptoms were also reported and included neurological manifestations, such as headache and dizziness [5]. With the exponential growth of reported COVID-19 cases, the neurological complications reported became more common. In a review by Sheraton *et al.*, the authors discussed the various neurological manifestations reported in retrospective studies, systematic reviews, and case reports published in previous months. The possible signs, symptoms, and complications that were observed regarding the central nervous system (CNS) were: headache, dizziness, alteration of mental status and delirium, dysexecutive syndrome, ataxia, acute cerebrovascular attacks, seizures, acute encephalitis, infectious and para-infectious encephalopathy, meningitis. Both ischemic and hemorrhagic stroke types were seen, with ischemic strokes being more common [6]. We reviewed the literature for proposed pathophysiological mechanisms by which SARS-CoV-2 can lead to the neurological complications presented above. They can be grouped as follows:

• Direct infection injury – resulting from infection of neurons and glial cells through blood circulation and neuronal pathways [6–9];
• Indirect effects of the infection on the nervous system – through hypoxia, systemic inflammatory response, hypercoagulability, and endothelial lesions (through direct lesions of the endothelial cells caused by the virus) [6–8, 10].

There are six other members, besides SARS-CoV-2, of the pathogenic coronaviruses family that cause human disease. The data from various studies regarding these coronaviruses suggest an evident neurotropism [10]. The new coronavirus may not be any different in this regard. It has been proposed that SARS-CoV-2 could use the expression of angiotensin-converting enzyme-2 (ACE2) in nervous tissue to gain entry inside neurons and glial cells [8, 10]. Non-ACE2 pathways for the infection of neural cells cannot be excluded (such as direct endocytotic infection) [10].

The neuronal pathway refers to the entry of a neurotropic virus in the CNS through the infection of sensory or motor nerve endings [6]. An example of a neuronal pathway is through the olfactory nerves and the olfactory bulb via a direct trans-synaptic route. This mechanism has been shown to be true for other coronaviruses as well [6, 10] and can lead to neuronal damage without substantial inflammation [9, 11]. In mice, ablation of the olfactory bulb prevented the spread of mouse hepatitis virus (MHV), a coronavirus that is genetically related to human coronaviruses, to the brain tissue [9]. Netland *et al.* used mice transgenic for human ACE2 to show that SARS-CoV enters the brain primarily via the olfactory bulb [11]. It is hypothesized that the data could be extrapolated for SARS-CoV-2 [8, 9, 11].

The blood circulation pathway refers to the entry of a virus in the CNS through the blood-brain barrier (BBB). It is potentiated by an increase in the BBB's permeability through endothelial lesions and systemic inflammation [6]. Endothelial cells also express ACE2, which allegedly interacts with the virus. Damage to the endothelial lining can favor viral access to the brain [8, 10]. Endothelial lesions resulting from the virus binding to the ACE2 and the cytokines and chemokines released during the systemic inflammatory response could also potentially lead to a rupture of the capillaries accompanied by bleeding within the cerebral tissue. They could also lead to neuroinflammation that severely disturbs brain homeostasis and causes neuronal cell death [8, 10]. Hypoxia due to respiratory distress is also an essential factor that could potentially lead to cerebral vasodilatation, cell swelling, and edema or stroke [7, 12]. The significantly increased inflammatory response due to the cytokine storm caused by the SARS-CoV-2 could be one of the causes of abnormal blood coagulation and lead to ischemic strokes and other cardiovascular events [7, 13].

The pathophysiology of CNS involvement in COVID-19 is mostly hypothetical. It is based on previous experience with other coronaviruses and could potentially be used as an explanation for the neurological manifestations in COVID-19. We present the case of a patient who developed multiple cerebral lesions, both ischemic and hemorrhagic, after being diagnosed with COVID 19 without a clear pathophysiological mechanism, which required taking into account various differential diagnoses.

## Case Presentation

A 50-year old Caucasian male residing in an urban area with right laterality was admitted to our Neurology Department with the complaint of weakness of the right limbs and impairment of the speaking ability. The symptoms had an acute onset, 5 days prior to presentation to our department. Family history revealed that the patient’s father had type II diabetes mellitus and suffered a cerebral ischemic stroke at 52. The patient had been a smoker (3 packs per day for 30 years) but was abstinent for the last 8 years. He also had class III obesity (body mass index of 42 kg/m2^2^).

### Patient history

Nine days prior to developing neurological symptoms, the patient presented to the Emergency Department nearest to home with dry cough, myalgia, pronounced fatigue, shivers, and fever (up to 39°C). A real-time polymerase chain reaction (RT-PCR) test was performed from a nasal swab sample in order to test for COVID-19, and the test came back positive. The patient was admitted to the Infectious Diseases Unit. A computed tomography (CT) scan of the chest was performed. It revealed bilateral, relatively symmetrical areas with ground-glass attenuation. Notable laboratory findings showed a biological inflammatory syndrome consisting of elevated C-reactive protein (CRP) levels – 8.3 mg/l, upper limit (UL) <5 mg/l), elevated fibrinogen (470 mg/dl, UL<400mg/dl), total leukocytes within the normal range but with lymphopenia – 5.5%, reference range (RR) = 20–40%) and neutrophilia (90.3%, RR=50–70%). During this time, D-dimer levels and platelet count were within the normal range. According to the World Health Organization (WHO) criteria for the severity of COVID-19 infection [14], the patient had a moderate form of infection. The treatment regimen consisted of Lopinavir/Ritonavir, Hydroxychloroquine 800mg/day, Enoxaparin 6000 IU/day, and Paracetamol (up to 2g per day). O^2^ saturation levels remained >96%. During the following week, the symptoms subsided, as well as the biological inflammatory syndrome. After 6 days, another RT-PCR test was performed. It came back negative, and the patient was discharged on the seventh day.

Two days after discharge, the patient woke up with weakness in the right limbs and troubled speaking, associated with generalized headache, nausea, and vomiting. He was transported to the regional Emergency Department. The on-call neurologist examined the patient. On neurological examination, hemiparesis (with a score of 4/5 on the Medical Research Council’s scale (MRC scale), central face palsy, diminished deep tendon reflexes, and extensor plantar reflex regarding the right side were objectified, along with motor dysphasia.

**Figure 1.**
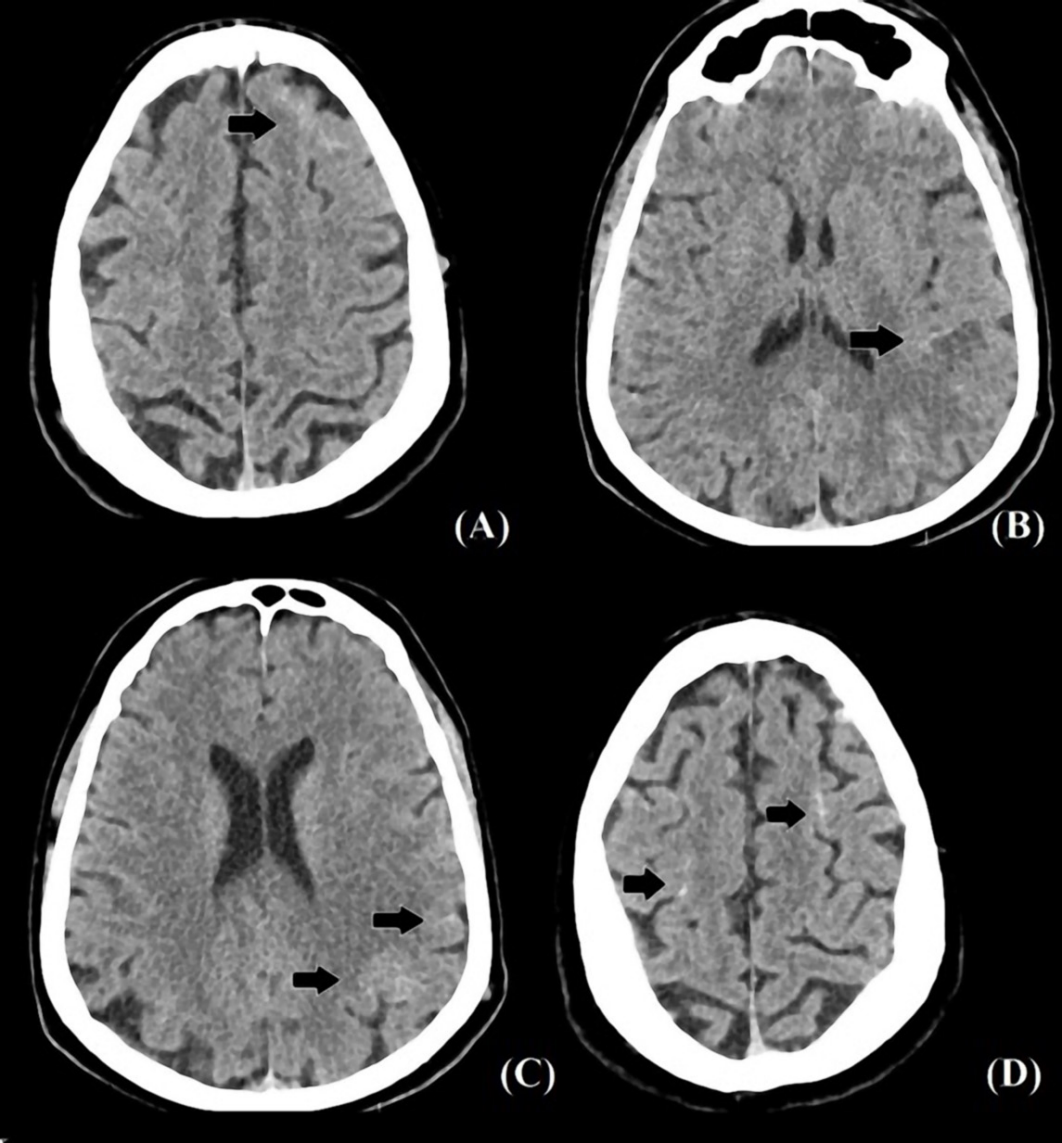
CT-scan, axial cross-sections of the brain. A, C, D - Arrows pointing to punctate subarachnoid hemorrhages. B - Arrow points to a small ischemic lesion with a narrow rim of hemorrhage.

**Figure 2.**
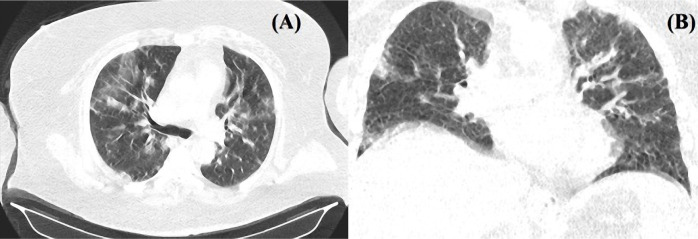
Chest CT. Axial cross-section (A) and coronal cross-section (B) showing diffuse areas with ground-glass attenuation.

A CT scan of the head without contrast medium was performed (about 6 hours after the patient noted the symptoms), but it did not show any abnormalities in the brain parenchyma. The bloodwork revealed mild leukocytosis (12.000/mm^3^, RR= 4.000–10.000/mm^3^) with neutrophilia (8.700/mm^3^, RR = 2.000–8000/mm^3^), elevated D-dimers (897.56 ng/ml, RR< 500 ng/ml). An acute cerebrovascular event was suspected, and the patient was started on dual antiplatelet therapy (Aspirin 75 mg per day + Clopidogrel 75 mg per day) and statin (Atorvastatin 40 mg per day). The blood pressure and heart rate were within the normal range. The electrocardiogram (ECG) was also normal. Thrombolysis was not indicated given the uncertainty regarding the moment of onset of the symptoms. A head CT without contrast medium was repeated (about 13 hours after the first one), along with a CT scan of the chest (Figures 1, 2).

The CT scan showed multiple hyperdense lesions, two of them located in the left frontal lobe (Figure 1A, Figure 1D), the right parietal lobe (Figure 1C), and the left parietal lobe (Figure 1D) that the radiologist interpreted as minimal subarachnoid hemorrhage. A small hypodense lesion with a narrow hyperdense rim was seen in the left temporoparietal region that was suggestive of an acute ischemic stroke with minimal hemorrhagic transformation (Figure 1B). The second chest CT was similar to the previous one, with bilateral relatively symmetrical areas with ground-glass attenuation (Figure 2).

Given the hemorrhagic nature of the lesions seen on the head CT (Figure 1), antiplatelet therapy was temporarily ceased. The patient received prophylactic Low-molecular-weight heparin (LMWH) – Enoxaparin 6000 IU/day and Paracetamol (up to 2g per day). An RT-PCR test was performed on the second day of admission with an equivocal result and another on the fourth day with a negative result. Nausea and vomiting did not persist after the first day. The intermittent frontal headache persisted during the time of admission, as well as the motor deficit and dysphasia, with a slight improvement. The patient was afterward transferred to our department, 5-days after the onset of the neurological symptoms.

On admission to our neurology hospital, the general and neurological examination and also hematological and biochemical parameters did not reveal any additional information compared to the prior examinations. Intermittent frontal headaches were persistent. The blood pressure (BP), heart rate, and O^2^ saturation levels were within the normal range. The patient was not dyspneic and had only mild coughing.

**Figure 3.**
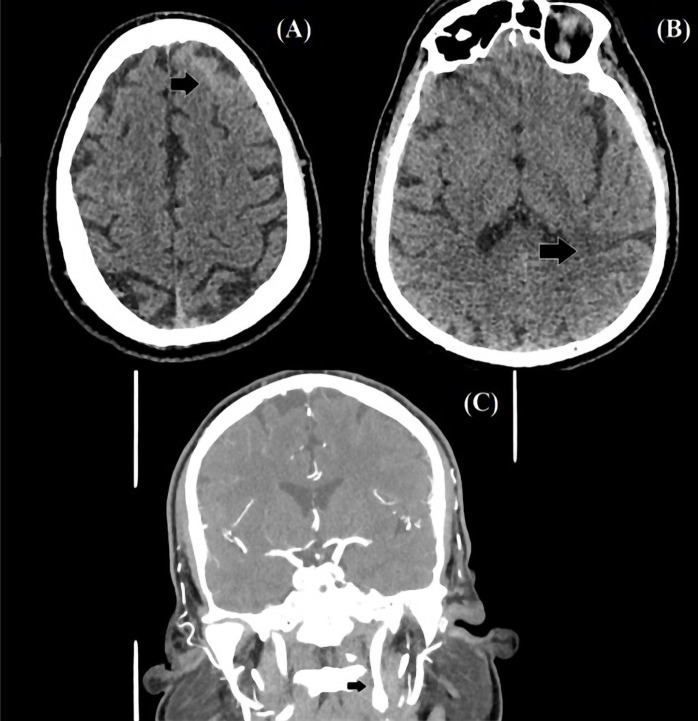
CT scan of the brain. Axial cross-section images showing the subarachnoid hemorrhage in the left frontal lobe (A) and the ischemic lesion in the left temporoparietal region (B). CT angiography, coronal cross-section, revealing a small filling defect in the cervical segment of the left internal carotid artery (C).

**Figure 4.**
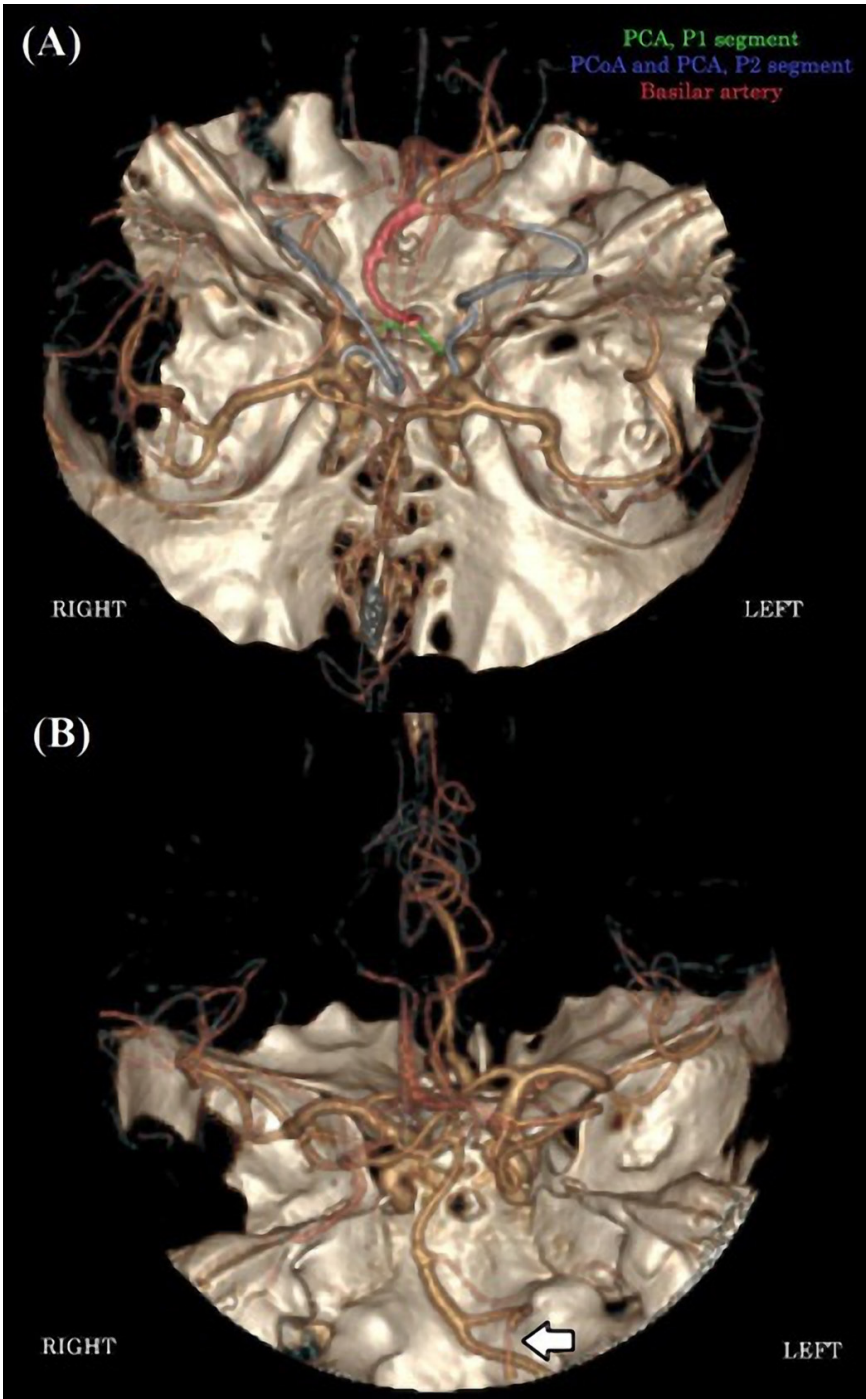
CT Angiography. Posterior circulation revealing both P1 segments of the posterior cerebral arteries are abnormally narrow (A). Arrow pointing to a tubular stenosis of the V4 segment of the left vertebral artery (B). PCA – Posterior cerebral artery; PCoA – Posterior communicating artery.

A head CT with angiography was performed 6 days after the onset of neurological symptoms. A slight improvement over the previous examination was seen regarding the subarachnoid and intraparenchymal hemorrhages (Figure 3A and 3B). A filling defect was observed in the C1 segment of the left internal carotid artery on angiography (Figure 3C) that was interpreted as partial thrombosis of the artery. Partial occlusion of the P1 segment of the right posterior cerebral artery, as well as narrowing of the P1 and P2 segments of the left posterior cerebral artery, were observed (Figure 4A). Tubular stenosis in the V4 segment of the left vertebral artery was also noted (Figure 4B).

A pulmonologist was consulted, and Dexamethasone 8 mg/day i.v. for 5 days with subsequent dose tapering was recommended, alongside prophylactic LMWH. Multiple ECG examinations were performed during the patient’s stay in the hospital, but none showed abnormalities.

**Figure 5.**
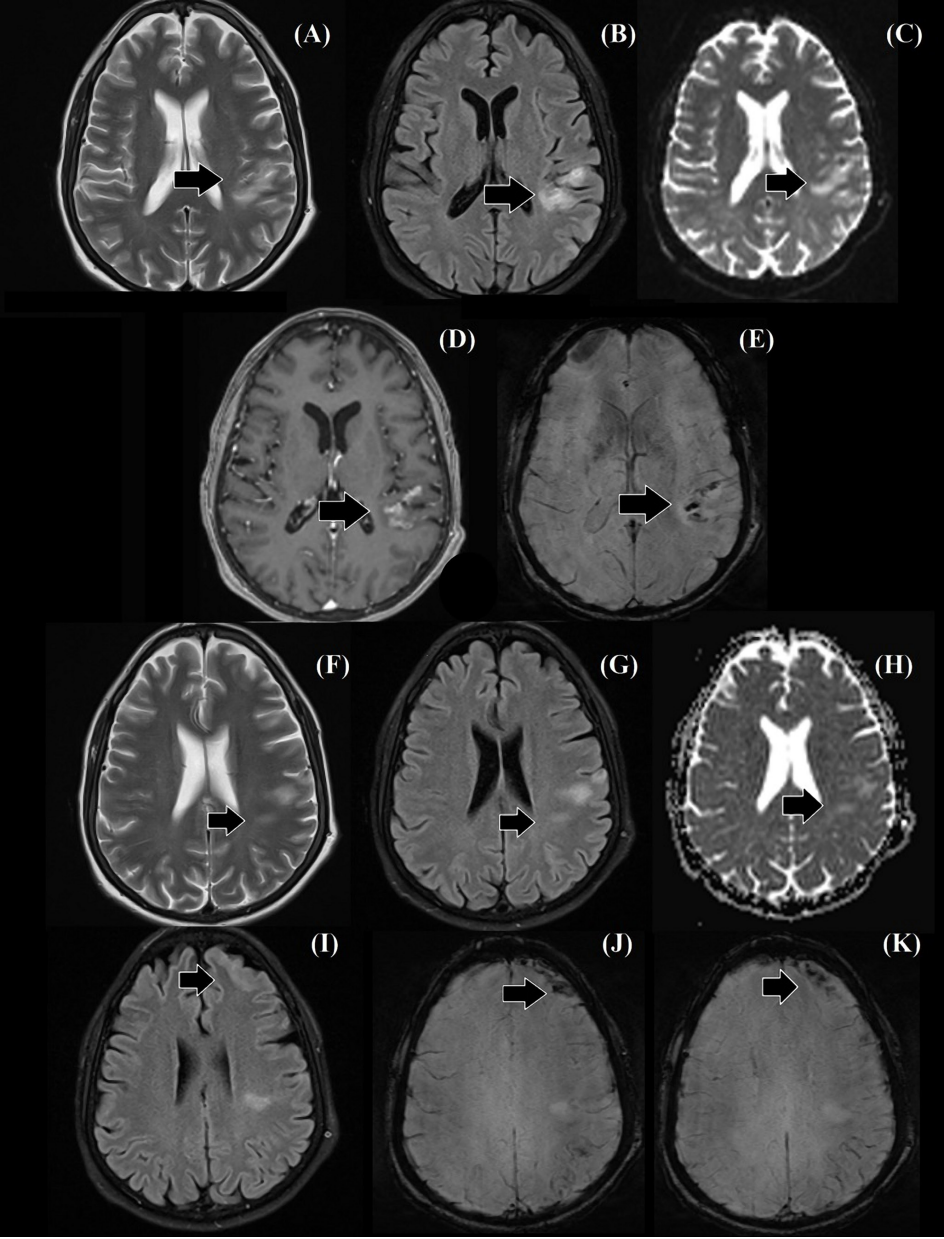
MRI scan of the brain, axial cross-section images. A–C) T2WI, FLAIR and DWI sequences showing the ischemic lesion in the left temporoparietal region. D, E) T1WI and SWI sequences revealing the hemorrhagic conversion of the lesion. F–H) T2WI, FLIAR and DWI sequences revealing a second ischemic lesion in the posterior area of the left parietal lobe. I–K) FLAIR and SWI sequences showing a convexal subarachnoid hemorrhage in the left frontal lobe. T2WI – T2 weighted image; FLAIR – Fluid attenuated inversion recovery; DWI – diffusion-weighted imaging.

A head magnetic resonance imaging (MRI) with contrast medium was performed 8 days after the onset of neurological symptoms to evaluate better the heterogeneous lesions in the brain parenchyma as well as the arterial and venous circulation. The cortico-subcortical temporoparietal lesion in the left hemisphere was compatible with a subacute ischemic stroke associated with minimal hemorrhagic transformation in the superficial territory of the left middle cerebral artery (Figure 5A-E). A similar, smaller lesion without any sign of hemorrhagic conversion was also observed in the left parietal lobe, posteriorly to the first one (Figure 5F-H). Minimal subarachnoid hemorrhage was described adjacent to the left frontal lobe (Figure 5I-K). The magnetic resonance angiography (MRA) revealed the same arterial lesions that were previously described on the computed tomography angiography (CTA): a small filling defect regarding the C1 segment of the left internal carotid artery as well as the stenoses of the left vertebral artery and the P1 segments of both posterior cerebral arteries. The MR-venogram did not show any abnormalities.

Given the heterogeneous nature of lesions present in our case and the plethora of atypical neurological complications in COVID-19 described in current literature (see the Discussion section), a lumbar puncture was performed to evaluate the possibility of encephalitis and cerebral vasculitis. Macroscopically, the cerebrospinal fluid (CSF) was clear. The biochemical and cytological examination was normal. Due to technical limitations, RT-PCR was not performed, so the presence of the SARS-Cov-2 genome in the CSF was not evaluated. The IgG index and the IgG ratio were normal as well. The serum biomarkers for autoimmune vasculitis were not present. CSF and serum analysis for various infectious pathogens that could cause encephalitis was performed, and we did not find any causative agents in our patient (Tables 1, 2). Thus, a diagnosis of encephalitis or cerebral vasculitis was considered to be unlikely.

**Table 1.** Multiplex PCR for Microbial Detection in Spinal Fluid (BIOFIRE FLMARRAY Panel).

Causative agent	Result
**Escherichia coli K1**	Undetectable
**Haemophilus influenzae**	Undetectable
**Listeria monocytogenes**	Undetectable
**Neisseria meningitidis**	Undetectable
**Streptococcus agalactiae**	Undetectable
**Streptococcus pneumoniae**	Undetectable
**Cytomegalovirus**	Undetectable
**Enterovirus**	Undetectable
**Herpes simplex virus 1**	Undetectable
**Herpes simplex virus 2**	Undetectable
**Herpes simplex virus 6**	Undetectable
**Parechovirus**	Undetectable
**Varicella Zoster virus**	Undetectable
**Cryptococus neoformans/gattii**	Undetectable

**Table 2.** Serum antibodies (IgG and IgM) for various causative agents.

Causative agent	IgM antibodies	IgG antibodies
**Herpes simplex virus 1**	Negative	Positive
**Herpes simplex virus 2**	Negative	Negative
**Epstein – Barr virus**	Negative	Positive
**Cytomegalovirus**	Negative	Positive
**Varicella Zoster virus**	Negative	Positive
**Toxoplasma gondii**	Negative	Positive
**Borrelia burgdorferi**	Negative	Negative

The clinical evolution was favorable during his 7-day stay in our hospital. The headaches disappeared after a few days. The motor deficit and dysphasia showed improvement as well. No abnormal laboratory results were present at the moment of discharge. The patient was referred to a cardiologist, a kinesiotherapist, and a logopedist after discharge, and the treatment plan consisted of Enoxaparin 6000 UI/day for a month and Aspirin 75 mg/day. A neurological re-examination (clinical and imaging) was scheduled one month later.

## Discussion

The case we presented raised multiple questions and possibilities regarding the differential diagnosis and the pathophysiological mechanism which could have been responsible for the lesions. There are a few pathologies that can lead to concomitant multifocal subarachnoid bleeds and ischemic strokes and could potentially be associated with a viral infection, such as COVID-19.

Ischemic strokes in the setting of COVID-19 have been described in recent literature and seem to be relatively frequent. In a study published by Li *et al.*, 10 out of 219 patients hospitalized for a COVID-19 infection developed an ischemic stroke [13]. The median time span from the first symptoms of SARS-CoV-2 infection to cerebrovascular disease was 10 days (ranging from 1 to 29). There are other studies that have also reported acute cerebrovascular events amongst these patients, and the severity of COVID-19 varied from mild to severe illness [15, 16]. It has been proposed that any acute infection could elicit an inflammatory activation in the plaque that could potentially lead to ischemic atherothrombotic events. Given the fact that COVID-19 also interacts with endothelial cells through the ACE receptors, this mechanism seems plausible. As previously mentioned, our patient also had a filling defect on the left internal carotid artery that could be interpreted in this setting [17, 18]. The possibility of a cardioembolic event was also discussed. There is emerging evidence that COVID-19 can associate with myocarditis [19], which could lead to cardioembolic events through various mechanisms (e.g., atrial fibrillation). Multiple ECG examinations were performed during admission, and although they were normal, paroxysmal arrhythmias cannot be excluded. The patient was referred to a cardiologist immediately after discharge for a Holter-ECG and an echocardiogram. A short note should be made regarding the D-dimer levels dynamic seen in our patient. D-dimers can increase during a COVID-19 infection, and abnormal levels could potentially indicate a poor prognosis [20]. The levels seen in our patient were normal until he developed neurological symptoms. Thus, it is more probable that the increase was due to the thrombus presence in a cerebral artery.

Unfortunately, the aspect discussed above did not explain the subarachnoid bleeds associated, and other possible pathologies were thus taken into account.

Given the presence of headache alongside neurological deficits, the imaging features, and the cerebral territories affected, we raised the suspicion of multiple cerebral venous thromboses (CVT). It has been proposed that SARS-CoV-2 could predispose to endothelial lesions and hypercoagulability, which could in turn potentially lead to CVT [18, 21, 22]. Numerous cases of CVT in patients infected with SARS-CoV-2 were reported in the literature [23–28]. Shakibajahromi *et al.* suggested that the presence of any unexplained and atypical hemorrhagic lesion in the initial brain CT of COVID-19 patients should raise the suspicion of CVT [27]. Oppenheim *et al.* also concluded that CVT should be considered when SAH is present, and the basal cistern is not involved [29], even if the subarachnoid hemorrhage is infrequent in the setting of a CVT [30]. An ischemic lesion, sometimes with a hemorrhagic component that crosses usual arterial boundaries or in close proximity to a venous sinus, is suggestive of CVT [30]. Our MRI and MR-venography excluded this possibility; however, any clinician should take into account the possibility of CVT when a COVID-19 patient presents with similar symptoms.

Two other similar pathologies we took into account, in terms of clinical features and imaging particularities, were primary angiitis of the central nervous system and the reversible cerebral vasoconstriction syndrome [31, 32]. Both pathologies could potentially be associated with infectious events and can cause ischemic strokes and small subarachnoid hemorrhage (SAH). We have also found in the literature a couple of case reports of patients who developed primary angiitis of the central nervous system (PACNS) [33] or reversible cerebral vasoconstriction syndrome (RCVS) [34] during or after COVID-19. Dakay *et al.* even proposed a mechanism through which SARS-CoV-2 could lead to RCVS and SAH. By interacting with endothelial cells and ACE2 receptors, it could cause dynamic vessel wall changes, vasoconstriction followed by vasodilation, leading to reperfusion injury and hemorrhage [34]. There are a few differentiating factors regarding RCVS and PACNS. RCVS usually present with severe thunderclap headaches, while PACNS patients have progressive, dull headaches [31, 32]. Our patient had moderate, intermittent headaches that subsided after a few days. However, in rare cases, headaches in RCVS can be mild or even absent [32]. We could hypothesize that cases with mild or moderate headaches could be more frequent, given the fact that RCVS is often misdiagnosed and a major criterion for establishing the diagnosis is the presence of severe, acute headaches [31, 32]. On the other hand, CSF examination is usually normal in RCVS and abnormal in >95% of PACNS patients [31, 32]. As we stated above, the CSF analysis was normal in the case of our patient. The CT and MR angiographies (performed 6–8 days after the onset of neurological symptoms) revealed some abnormalities concerning a segment of the left vertebral artery and both posterior cerebral arteries (P1 segments). In RCVS, the abnormalities on neurovascular imaging are present in the acute stage and are reversible within days to weeks, while those in PACNS are frequently irreversible [31, 32]. Nevertheless, in our patient, the stenoses present on the P1 segment of the posterior cerebral arteries could be a congenital variant. These aspects remain to be evaluated at the 1-month follow-up. Therefore, we could not rule out either of these diagnoses, and they both remain possible, albeit somewhat improbable.

Last but not least, there have been cases of SARS-CoV-2-associated encephalitis and encephalopathy, with various atypical clinical manifestations and imaging features, in recent publications [35–40]. Moriguchi *et al.* presented a case of meningitis/encephalitis with a positive RT-PCR test for SARS-CoV-2 from the CSF, thus showing the neuro-invasive potential of the virus [38]. Zuhorn *et al.* presented a case of parainfectious encephalitis that they believed was of autoimmune nature, given that their patient had a negative RT-PCR test from the CSF [40]. Paniz *et al.* hypothesized that failure to detect the virus in the CSF in their case and others could have several explanations. The first potential explanation is that the virus is mainly cell-bound, spreading from cell to cell. Another possibility is that the virus may be at concentrations below the level of detection of the testing method and a third possibility is the presence of low concentrations of endonucleases/exonucleases and proteins acting as inhibitors in the CSF [18]. Patel *et al.* published a case presentation of a Varicella-zoster virus encephalitis associated with the COVID-19 infection, underlying the fact that COVID-19 could lead to an immunosuppressed state (due to the infection itself or immunosuppressant drugs) that allows other pathogens to reactivate (41). As we stated before, we could not evaluate the presence of SARS-CoV-2 in the CSF of our patient due to technical limitations. However, given the unspecific and equivocal neurological findings in patients with COVID-19, we prompt our readers to be diligent when evaluating such patients.

## Conclusion

The spectrum of neurological complications in COVID-19 is not yet sufficiently understood and remains to be discovered and explained through more extensive studies. Still, the limited knowledge at this time should not cloud the clinical judgment but determine clinicians to fully investigate their patients, be diligent in evaluating current literature, and report their findings, even if a conclusive, positive diagnosis could not be established. Our case proved to be challenging in terms of differential diagnosis, and we hope that future research will shed light on the pathophysiological mechanisms through which SARS-CoV-2 interacts with and affects the nervous system.

## Acknowledgments

The authors would like to thank Ioana Robu from the Iuliu Hatieganu University of Medicine and Farmacy of Cluj, Romania, for her help in editing this manuscript.

### Conflict of interest

The authors declare that there is no conflict of interest.
